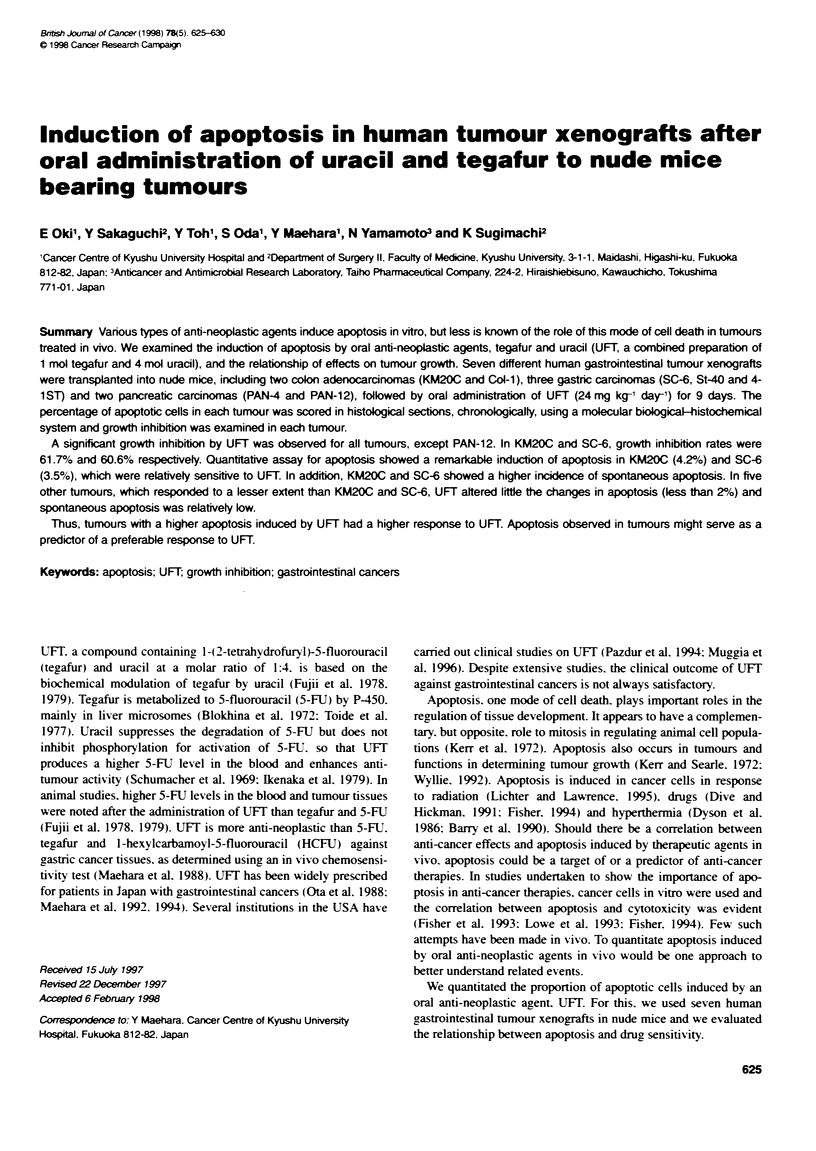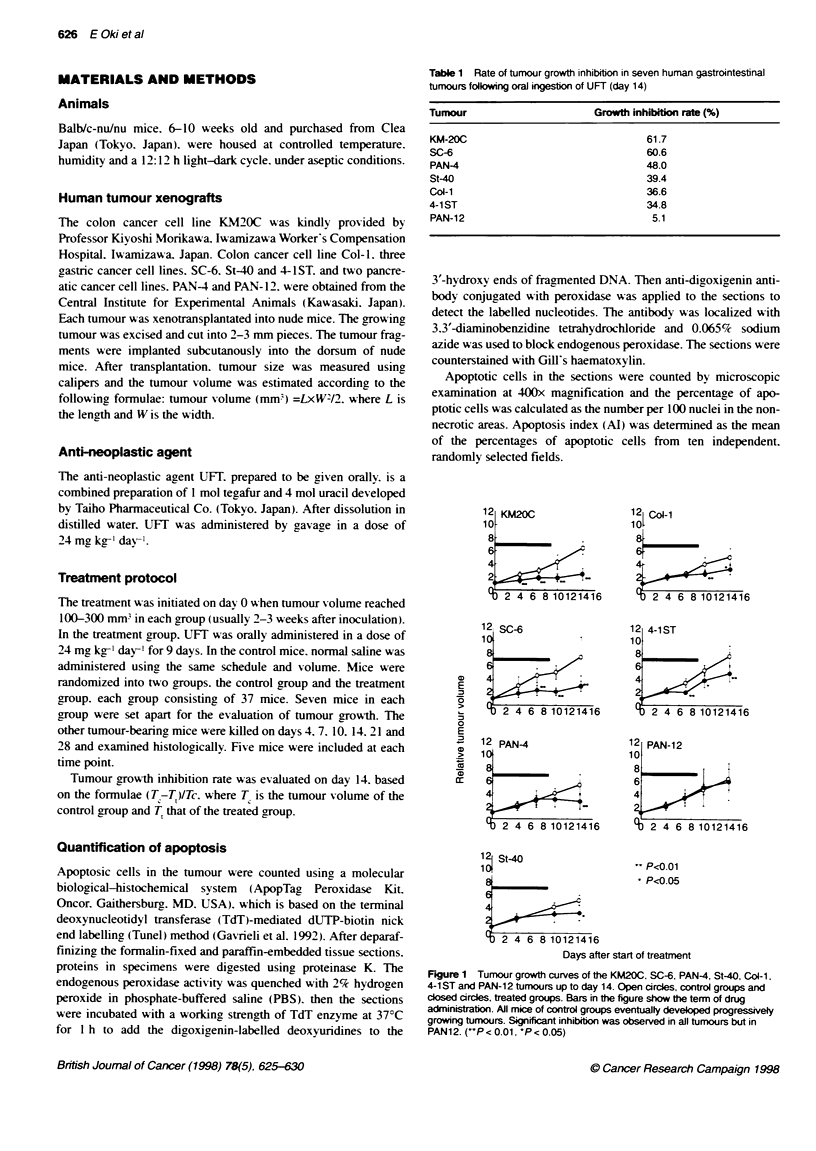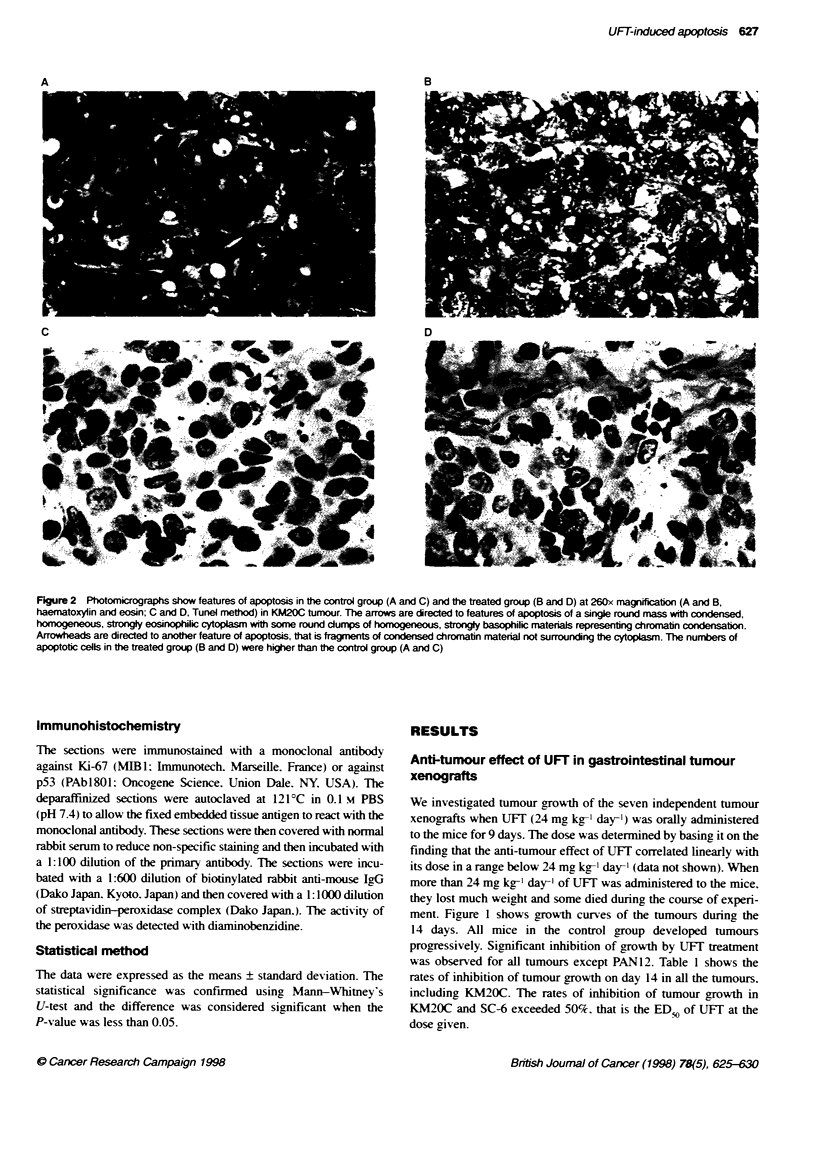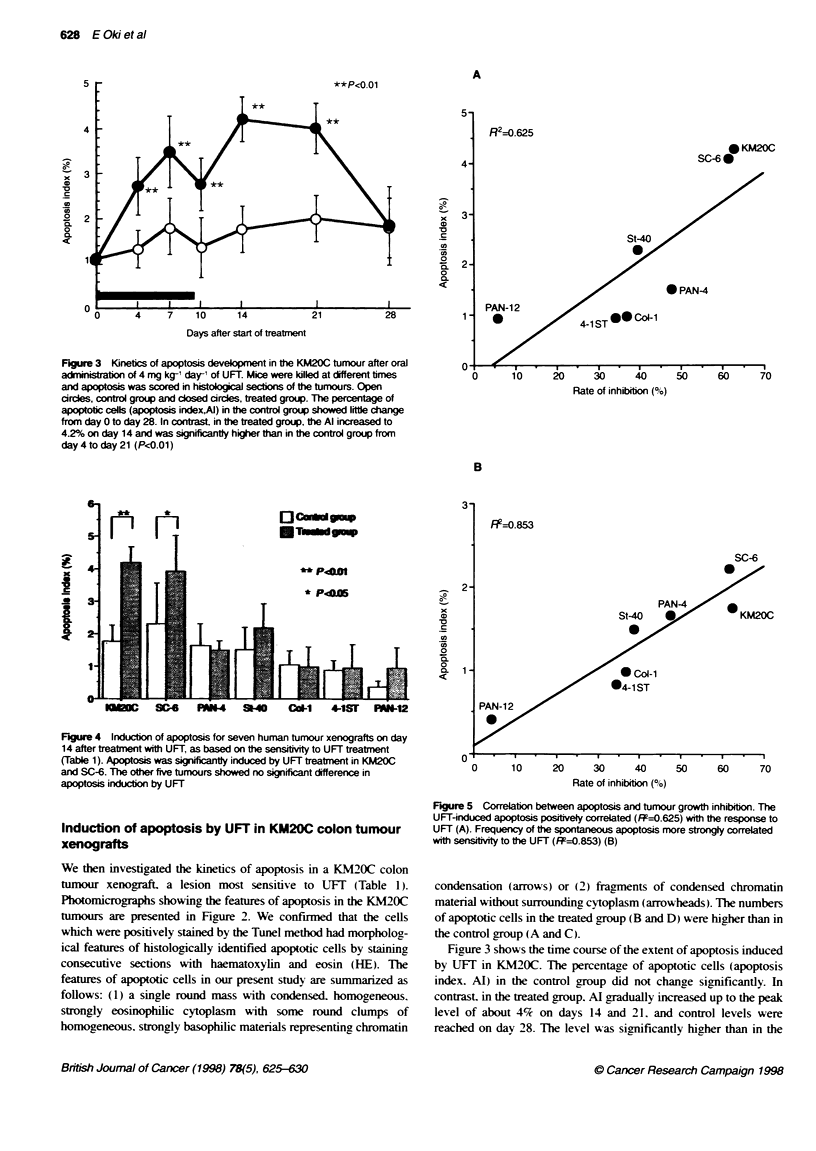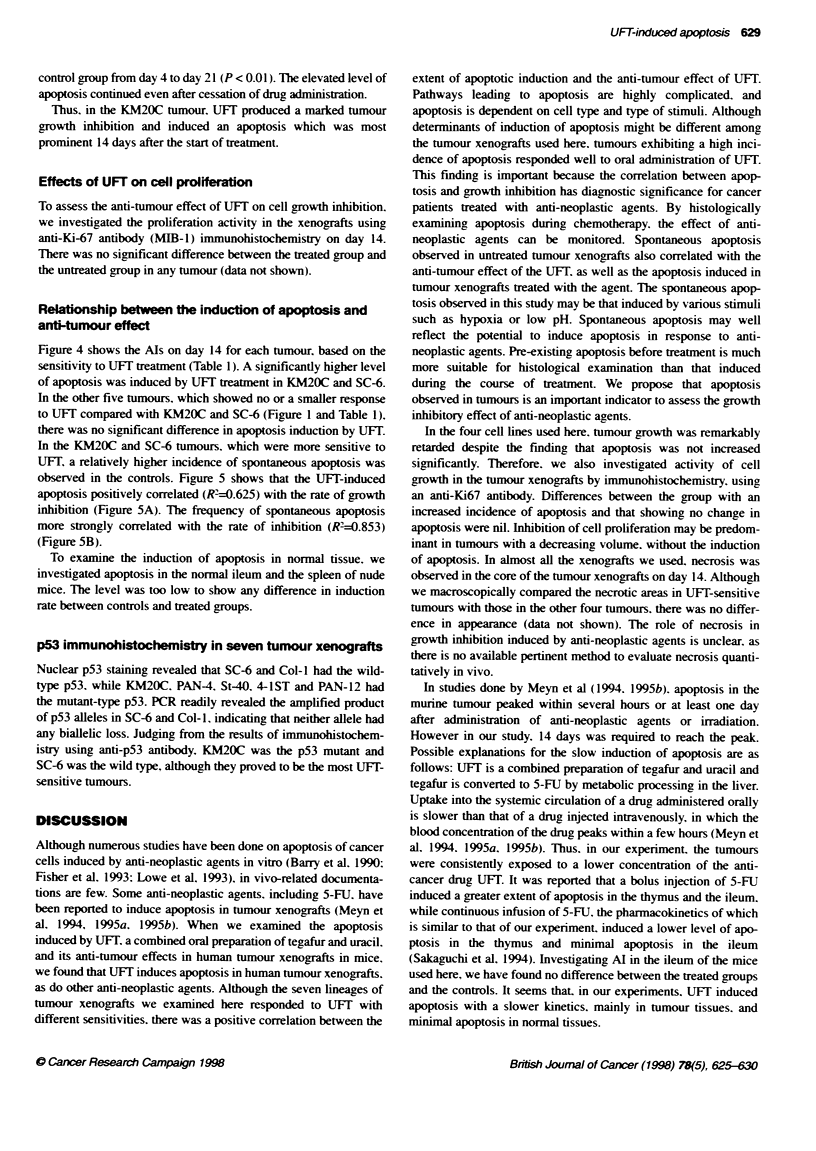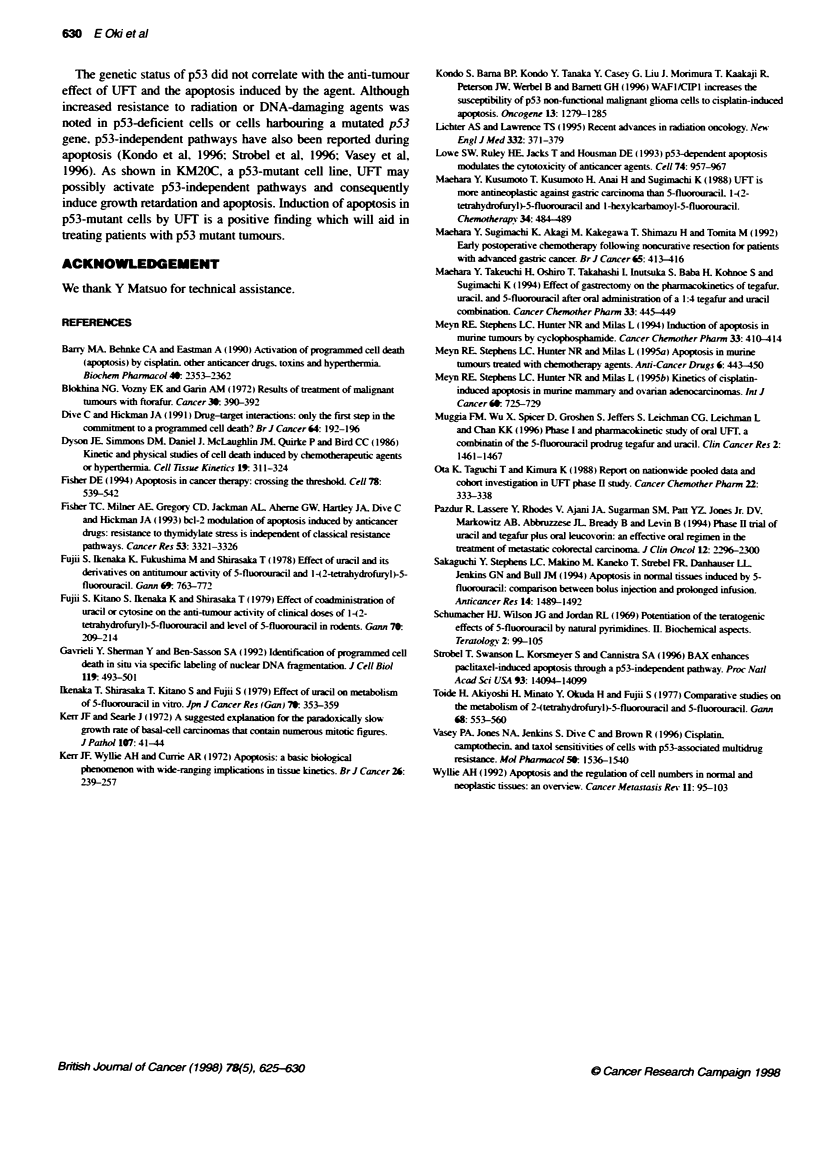# Induction of apoptosis in human tumour xenografts after oral administration of uracil and tegafur to nude mice bearing tumours.

**DOI:** 10.1038/bjc.1998.552

**Published:** 1998-09

**Authors:** E. Oki, Y. Sakaguchi, Y. Toh, S. Oda, Y. Maehara, N. Yamamoto, K. Sugimachi

**Affiliations:** Cancer Centre of Kyushu University Hospital, Fukuoka, Japan.

## Abstract

**Images:**


British Journal of Cancer (1998) 78(5). 625–630
© 1998 Cancer Research Campaign

### Induction of apoptosis in human tumour xenografts after oral administration of uracil and tegafur to nude mice bearing tumours

E Oki[1], Y Sakaguchi[2], Y Toh[1], S Oda[1], Y Maehara[1], N Yamamoto[3] and K Sugimachi[2]

[1]Cancer Centre of Kyushu University Hospital and [2]Department of Surgery II, Faculty of Medicine, Kyushu University, 3-1-1, Maidashi, Higashi-ku, Fukuoka 812-82, Japan; [3]Anticancer and Antimicrobial Research Laboratory, Taiho Pharmaceutical Company, 224-2, Hiraishiebisuno, Kawauchicho, Tokushima 771-01, Japan

**Summary** Various types of anti-neoplastic agents induce apoptosis in vitro, but less is known of the role of this mode of cell death in tumours treated in vivo. We examined the induction of apoptosis by oral anti-neoplastic agents, tegafur and uracil (UFT, a combined preparation of 1 mol tegafur and 4 mol uracil), and the relationship of effects on tumour growth. Seven different human gastrointestinal tumour xenografts were transplanted into nude mice, including two colon adenocarcinomas (KM20C and Col-1), three gastric carcinomas (SC-6, St-40 and 4-1ST) and two pancreatic carcinomas (PAN-4 and PAN-12), followed by oral administration of UFT (24 mg $kg^{-1}$ $day^{-1}$) for 9 days. The percentage of apoptotic cells in each tumour was scored in histological sections, chronologically, using a molecular biological–histochemical system and growth inhibition was examined in each tumour.

A significant growth inhibition by UFT was observed for all tumours, except PAN-12. In KM20C and SC-6, growth inhibition rates were 61.7% and 60.6% respectively. Quantitative assay for apoptosis showed a remarkable induction of apoptosis in KM20C (4.2%) and SC-6 (3.5%), which were relatively sensitive to UFT. In addition, KM20C and SC-6 showed a higher incidence of spontaneous apoptosis. In five other tumours, which responded to a lesser extent than KM20C and SC-6, UFT altered little the changes in apoptosis (less than 2%) and spontaneous apoptosis was relatively low.

Thus, tumours with a higher apoptosis induced by UFT had a higher response to UFT. Apoptosis observed in tumours might serve as a predictor of a preferable response to UFT.

**Keywords:** apoptosis; UFT; growth inhibition; gastrointestinal cancers

UFT, a compound containing 1-(2-tetrahydrofuryl)-5-fluorouracil (tegafur) and uracil at a molar ratio of 1:4, is based on the biochemical modulation of tegafur by uracil (Fujii et al, 1978, 1979). Tegafur is metabolized to 5-fluorouracil (5-FU) by P-450, mainly in liver microsomes (Blokhina et al, 1972; Toide et al, 1977). Uracil suppresses the degradation of 5-FU but does not inhibit phosphorylation for activation of 5-FU, so that UFT produces a higher 5-FU level in the blood and enhances anti-tumour activity (Schumacher et al, 1969; Ikenaka et al, 1979). In animal studies, higher 5-FU levels in the blood and tumour tissues were noted after the administration of UFT than tegafur and 5-FU (Fujii et al, 1978, 1979). UFT is more anti-neoplastic than 5-FU, tegafur and 1-hexylcarbamoyl-5-fluorouracil (HCFU) against gastric cancer tissues, as determined using an in vivo chemosensitivity test (Maehara et al, 1988). UFT has been widely prescribed for patients in Japan with gastrointestinal cancers (Ota et al, 1988; Maehara et al, 1992, 1994). Several institutions in the USA have carried out clinical studies on UFT (Pazdur et al, 1994; Muggia et al, 1996). Despite extensive studies, the clinical outcome of UFT against gastrointestinal cancers is not always satisfactory.

Apoptosis, one mode of cell death, plays important roles in the regulation of tissue development. It appears to have a complementary, but opposite, role to mitosis in regulating animal cell populations (Kerr et al, 1972). Apoptosis also occurs in tumours and functions in determining tumour growth (Kerr and Searle, 1972; Wyllie, 1992). Apoptosis is induced in cancer cells in response to radiation (Lichter and Lawrence, 1995), drugs (Dive and Hickman, 1991; Fisher, 1994) and hyperthermia (Dyson et al, 1986; Barry et al, 1990). Should there be a correlation between anti-cancer effects and apoptosis induced by therapeutic agents in vivo, apoptosis could be a target of or a predictor of anti-cancer therapies. In studies undertaken to show the importance of apoptosis in anti-cancer therapies, cancer cells in vitro were used and the correlation between apoptosis and cytotoxicity was evident (Fisher et al, 1993; Lowe et al, 1993; Fisher, 1994). Few such attempts have been made in vivo. To quantitate apoptosis induced by oral anti-neoplastic agents in vivo would be one approach to better understand related events.

We quantitated the proportion of apoptotic cells induced by an oral anti-neoplastic agent, UFT. For this, we used seven human gastrointestinal tumour xenografts in nude mice and we evaluated the relationship between apoptosis and drug sensitivity.

*Received 15 July 1997*
*Revised 22 December 1997*
*Accepted 6 February 1998*

*Correspondence to:* Y Maehara, Cancer Centre of Kyushu University Hospital, Fukuoka 812-82, Japan

### MATERIALS AND METHODS

### Animals

Balb/c-nu/nu mice, 6–10 weeks old and purchased from Clea Japan (Tokyo, Japan), were housed at controlled temperature, humidity and a 12:12 h light–dark cycle, under aseptic conditions.

### Human tumour xenografts

The colon cancer cell line KM20C was kindly provided by Professor Kiyoshi Morikawa, Iwamizawa Worker's Compensation Hospital, Iwamizawa, Japan. Colon cancer cell line Col-1, three gastric cancer cell lines, SC-6, St-40 and 4-1ST, and two pancreatic cancer cell lines, PAN-4 and PAN-12, were obtained from the Central Institute for Experimental Animals (Kawasaki, Japan). Each tumour was xenotransplantated into nude mice. The growing tumour was excised and cut into 2–3 mm pieces. The tumour fragments were implanted subcutanously into the dorsum of nude mice. After transplantation, tumour size was measured using calipers and the tumour volume was estimated according to the following formulae: tumour volume ($mm^3$) $=L \times W^2/2$, where $L$ is the length and $W$ is the width.

### Anti-neoplastic agent

The anti-neoplastic agent UFT, prepared to be given orally, is a combined preparation of 1 mol tegafur and 4 mol uracil developed by Taiho Pharmaceutical Co. (Tokyo, Japan). After dissolution in distilled water, UFT was administered by gavage in a dose of 24 mg $kg^{-1}$ $day^{-1}$.

### Treatment protocol

The treatment was initiated on day 0 when tumour volume reached 100–300 $mm^3$ in each group (usually 2–3 weeks after inoculation). In the treatment group, UFT was orally administered in a dose of 24 mg $kg^{-1}$ $day^{-1}$ for 9 days. In the control mice, normal saline was administered using the same schedule and volume. Mice were randomized into two groups, the control group and the treatment group, each group consisting of 37 mice. Seven mice in each group were set apart for the evaluation of tumour growth. The other tumour-bearing mice were killed on days 4, 7, 10, 14, 21 and 28 and examined histologically. Five mice were included at each time point.

Tumour growth inhibition rate was evaluated on day 14, based on the formulae $(T_c-T_t)/Tc$, where $T_c$ is the tumour volume of the control group and $T_t$ that of the treated group.

### Quantification of apoptosis

Apoptosic cells in the tumour were counted using a molecular biological–histochemical system (ApopTag Peroxidase Kit, Oncor, Gaithersburg, MD, USA), which is based on the terminal deoxynucleotidyl transferase (TdT)-mediated dUTP-biotin nick end labelling (Tunel) method (Gavrieli et al, 1992). After deparaffinizing the formalin-fixed and paraffin-embedded tissue sections, proteins in specimens were digested using proteinase K. The endogenous peroxidase activity was quenched with 2% hydrogen peroxide in phosphate-buffered saline (PBS), then the sections were incubated with a working strength of TdT enzyme at 37°C for 1 h to add the digoxigenin-labelled deoxyuridines to the 3′-hydroxy ends of fragmented DNA. Then anti-digoxigenin antibody conjugated with peroxidase was applied to the sections to detect the labelled nucleotides. The antibody was localized with 3,3′-diaminobenzidine tetrahydrochloride and 0.065% sodium azide was used to block endogenous peroxidase. The sections were counterstained with Gill's haematoxylin.

Apoptotic cells in the sections were counted by microscopic examination at 400× magnification and the percentage of apoptotic cells was calculated as the number per 100 nuclei in the non-necrotic areas. Apoptosis index (AI) was determined as the mean of the percentages of apoptotic cells from ten independent, randomly selected fields.

**Table 1** Rate of tumour growth inhibition in seven human gastrointestinal tumours following oral ingestion of UFT (day 14)

| Tumour | Growth inhibition rate (%) |
|---|---|
| KM-20C | 61.7 |
| SC-6 | 60.6 |
| PAN-4 | 48.0 |
| St-40 | 39.4 |
| Col-1 | 36.6 |
| 4-1ST | 34.8 |
| PAN-12 | 5.1 |

**Figure 1** Tumour growth curves of the KM20C, SC-6, PAN-4, St-40, Col-1, 4-1ST and PAN-12 tumours up to day 14. Open circles, control groups and closed circles, treated groups. Bars in the figure show the term of drug administration. All mice of control groups eventually developed progressively growing tumours. Significant inhibition was observed in all tumours but in PAN12. (**$P < 0.01$, *$P < 0.05$)

© Cancer Research Campaign 1998

**Figure 2** Photomicrographs show features of apoptosis in the control group (A and C) and the treated group (B and D) at 260× magnification (A and B, haematoxylin and eosin; C and D, Tunel method) in KM20C tumour. The arrows are directed to features of apoptosis of a single round mass with condensed, homogeneous, strongly eosinophilic cytoplasm with some round clumps of homogeneous, strongly basophilic materials representing chromatin condensation. Arrowheads are directed to another feature of apoptosis, that is fragments of condensed chromatin material not surrounding the cytoplasm. The numbers of apoptotic cells in the treated group (B and D) were higher than the control group (A and C)

### Immunohistochemistry

The sections were immunostained with a monoclonal antibody against Ki-67 (MIB1: Immunotech, Marseille, France) or against p53 (PAb1801: Oncogene Science, Union Dale, NY, USA). The deparaffinized sections were autoclaved at 121°C in 0.1 M PBS (pH 7.4) to allow the fixed embedded tissue antigen to react with the monoclonal antibody. These sections were then covered with normal rabbit serum to reduce non-specific staining and then incubated with a 1:100 dilution of the primary antibody. The sections were incubated with a 1:600 dilution of biotinylated rabbit anti-mouse IgG (Dako Japan, Kyoto, Japan) and then covered with a 1:1000 dilution of streptavidin–peroxidase complex (Dako Japan,). The activity of the peroxidase was detected with diaminobenzidine.

### Statistical method

The data were expressed as the means ± standard deviation. The statistical significance was confirmed using Mann–Whitney's *U*-test and the difference was considered significant when the *P*-value was less than 0.05.

### RESULTS

### Anti-tumour effect of UFT in gastrointestinal tumour xenografts

We investigated tumour growth of the seven independent tumour xenografts when UFT (24 mg $kg^{-1}$ $day^{-1}$) was orally administered to the mice for 9 days. The dose was determined by basing it on the finding that the anti-tumour effect of UFT correlated linearly with its dose in a range below 24 mg $kg^{-1}$ $day^{-1}$ (data not shown). When more than 24 mg $kg^{-1}$ $day^{-1}$ of UFT was administered to the mice, they lost much weight and some died during the course of experiment. Figure 1 shows growth curves of the tumours during the 14 days. All mice in the control group developed tumours progressively. Significant inhibition of growth by UFT treatment was observed for all tumours except PAN12. Table 1 shows the rates of inhibition of tumour growth on day 14 in all the tumours, including KM20C. The rates of inhibition of tumour growth in KM20C and SC-6 exceeded 50%, that is the $ED_{50}$ of UFT at the dose given.

© Cancer Research Campaign 1998

**Figure 3** Kinetics of apoptosis development in the KM20C tumour after oral administration of 4 mg kg−1 day−1 of UFT. Mice were killed at different times and apoptosis was scored in histological sections of the tumours. Open circles, control group and closed circles, treated group. The percentage of apoptotic cells (apoptosis index,AI) in the control group showed little change from day 0 to day 28. In contrast, in the treated group, the AI increased to 4.2% on day 14 and was significantly higher than in the control group from day 4 to day 21 (*P*<0.01)

**Figure 4** Induction of apoptosis for seven human tumour xenografts on day 14 after treatment with UFT, as based on the sensitivity to UFT treatment (Table 1). Apoptosis was significantly induced by UFT treatment in KM20C and SC-6. The other five tumours showed no significant difference in apoptosis induction by UFT

**Figure 5** Correlation between apoptosis and tumour growth inhibition. The UFT-induced apoptosis positively correlated ($R^2$=0.625) with the response to UFT (A). Frequency of the spontaneous apoptosis more strongly correlated with sensitivity to the UFT ($R^2$=0.853) (B)

### Induction of apoptosis by UFT in KM20C colon tumour xenografts

We then investigated the kinetics of apoptosis in a KM20C colon tumour xenograft, a lesion most sensitive to UFT (Table 1). Photomicrographs showing the features of apoptosis in the KM20C tumours are presented in Figure 2. We confirmed that the cells which were positively stained by the Tunel method had morphological features of histologically identified apoptotic cells by staining consecutive sections with haematoxylin and eosin (HE). The features of apoptotic cells in our present study are summarized as follows: (1) a single round mass with condensed, homogeneous, strongly eosinophilic cytoplasm with some round clumps of homogeneous, strongly basophilic materials representing chromatin condensation (arrows) or (2) fragments of condensed chromatin material without surrounding cytoplasm (arrowheads). The numbers of apoptotic cells in the treated group (B and D) were higher than in the control group (A and C).

Figure 3 shows the time course of the extent of apoptosis induced by UFT in KM20C. The percentage of apoptotic cells (apoptosis index, AI) in the control group did not change significantly. In contrast, in the treated group, AI gradually increased up to the peak level of about 4% on days 14 and 21, and control levels were reached on day 28. The level was significantly higher than in the

© Cancer Research Campaign 1998

control group from day 4 to day 21 ($P < 0.01$). The elevated level of apoptosis continued even after cessation of drug administration.

Thus, in the KM20C tumour, UFT produced a marked tumour growth inhibition and induced an apoptosis which was most prominent 14 days after the start of treatment.

### Effects of UFT on cell proliferation

To assess the anti-tumour effect of UFT on cell growth inhibition, we investigated the proliferation activity in the xenografts using anti-Ki-67 antibody (MIB-1) immunohistochemistry on day 14. There was no significant difference between the treated group and the untreated group in any tumour (data not shown).

### Relationship between the induction of apoptosis and anti-tumour effect

Figure 4 shows the AIs on day 14 for each tumour, based on the sensitivity to UFT treatment (Table 1). A significantly higher level of apoptosis was induced by UFT treatment in KM20C and SC-6. In the other five tumours, which showed no or a smaller response to UFT compared with KM20C and SC-6 (Figure 1 and Table 1), there was no significant difference in apoptosis induction by UFT. In the KM20C and SC-6 tumours, which were more sensitive to UFT, a relatively higher incidence of spontaneous apoptosis was observed in the controls. Figure 5 shows that the UFT-induced apoptosis positively correlated ($R^2$=0.625) with the rate of growth inhibition (Figure 5A). The frequency of spontaneous apoptosis more strongly correlated with the rate of inhibition ($R^2$=0.853) (Figure 5B).

To examine the induction of apoptosis in normal tissue, we investigated apoptosis in the normal ileum and the spleen of nude mice. The level was too low to show any difference in induction rate between controls and treated groups.

### p53 immunohistochemistry in seven tumour xenografts

Nuclear p53 staining revealed that SC-6 and Col-1 had the wild-type p53, while KM20C, PAN-4, St-40, 4-1ST and PAN-12 had the mutant-type p53. PCR readily revealed the amplified product of p53 alleles in SC-6 and Col-1, indicating that neither allele had any biallelic loss. Judging from the results of immunohistochemistry using anti-p53 antibody, KM20C was the p53 mutant and SC-6 was the wild type, although they proved to be the most UFT-sensitive tumours.

### DISCUSSION

Although numerous studies have been done on apoptosis of cancer cells induced by anti-neoplastic agents in vitro (Barry et al, 1990; Fisher et al, 1993; Lowe et al, 1993), in vivo-related documentations are few. Some anti-neoplastic agents, including 5-FU, have been reported to induce apoptosis in tumour xenografts (Meyn et al, 1994, 1995*a*, 1995*b*). When we examined the apoptosis induced by UFT, a combined oral preparation of tegafur and uracil, and its anti-tumour effects in human tumour xenografts in mice, we found that UFT induces apoptosis in human tumour xenografts, as do other anti-neoplastic agents. Although the seven lineages of tumour xenografts we examined here responded to UFT with different sensitivities, there was a positive correlation between the extent of apoptotic induction and the anti-tumour effect of UFT. Pathways leading to apoptosis are highly complicated, and apoptosis is dependent on cell type and type of stimuli. Although determinants of induction of apoptosis might be different among the tumour xenografts used here, tumours exhibiting a high incidence of apoptosis responded well to oral administration of UFT. This finding is important because the correlation between apoptosis and growth inhibition has diagnostic significance for cancer patients treated with anti-neoplastic agents. By histologically examining apoptosis during chemotherapy, the effect of anti-neoplastic agents can be monitored. Spontaneous apoptosis observed in untreated tumour xenografts also correlated with the anti-tumour effect of the UFT, as well as the apoptosis induced in tumour xenografts treated with the agent. The spontaneous apoptosis observed in this study may be that induced by various stimuli such as hypoxia or low pH. Spontaneous apoptosis may well reflect the potential to induce apoptosis in response to anti-neoplastic agents. Pre-existing apoptosis before treatment is much more suitable for histological examination than that induced during the course of treatment. We propose that apoptosis observed in tumours is an important indicator to assess the growth inhibitory effect of anti-neoplastic agents.

In the four cell lines used here, tumour growth was remarkably retarded despite the finding that apoptosis was not increased significantly. Therefore, we also investigated activity of cell growth in the tumour xenografts by immunohistochemistry, using an anti-Ki67 antibody. Differences between the group with an increased incidence of apoptosis and that showing no change in apoptosis were nil. Inhibition of cell proliferation may be predominant in tumours with a decreasing volume, without the induction of apoptosis. In almost all the xenografts we used, necrosis was observed in the core of the tumour xenografts on day 14. Although we macroscopically compared the necrotic areas in UFT-sensitive tumours with those in the other four tumours, there was no difference in appearance (data not shown). The role of necrosis in growth inhibition induced by anti-neoplastic agents is unclear, as there is no available pertinent method to evaluate necrosis quantitatively in vivo.

In studies done by Meyn et al (1994, 1995*b*), apoptosis in the murine tumour peaked within several hours or at least one day after administration of anti-neoplastic agents or irradiation. However in our study, 14 days was required to reach the peak. Possible explanations for the slow induction of apoptosis are as follows: UFT is a combined preparation of tegafur and uracil and tegafur is converted to 5-FU by metabolic processing in the liver. Uptake into the systemic circulation of a drug administered orally is slower than that of a drug injected intravenously, in which the blood concentration of the drug peaks within a few hours (Meyn et al, 1994, 1995*a*, 1995*b*). Thus, in our experiment, the tumours were consistently exposed to a lower concentration of the anti-cancer drug UFT. It was reported that a bolus injection of 5-FU induced a greater extent of apoptosis in the thymus and the ileum, while continuous infusion of 5-FU, the pharmacokinetics of which is similar to that of our experiment, induced a lower level of apoptosis in the thymus and minimal apoptosis in the ileum (Sakaguchi et al, 1994). Investigating AI in the ileum of the mice used here, we have found no difference between the treated groups and the controls. It seems that, in our experiments, UFT induced apoptosis with a slower kinetics, mainly in tumour tissues, and minimal apoptosis in normal tissues.

© Cancer Research Campaign 1998

The genetic status of p53 did not correlate with the anti-tumour effect of UFT and the apoptosis induced by the agent. Although increased resistance to radiation or DNA-damaging agents was noted in p53-deficient cells or cells harbouring a mutated *p53* gene, p53-independent pathways have also been reported during apoptosis (Kondo et al, 1996; Strobel et al, 1996; Vasey et al, 1996). As shown in KM20C, a p53-mutant cell line, UFT may possibly activate p53-independent pathways and consequently induce growth retardation and apoptosis. Induction of apoptosis in p53-mutant cells by UFT is a positive finding which will aid in treating patients with p53 mutant tumours.

### ACKNOWLEDGEMENT

We thank Y Matsuo for technical assistance.

### REFERENCES

Barry MA, Behnke CA and Eastman A (1990) Activation of programmed cell death (apoptosis) by cisplatin, other anticancer drugs, toxins and hyperthermia. *Biochem Pharmacol* **40**: 2353–2362

Blokhina NG, Vozny EK and Garin AM (1972) Results of treatment of malignant tumours with ftorafur. *Cancer* **30**: 390–392

Dive C and Hickman JA (1991) Drug–target interactions: only the first step in the commitment to a programmed cell death? *Br J Cancer* **64**: 192–196

Dyson JE, Simmons DM, Daniel J, McLaughlin JM, Quirke P and Bird CC (1986) Kinetic and physical studies of cell death induced by chemotherapeutic agents or hyperthermia. *Cell Tissue Kinetics* **19**: 311–324

Fisher DE (1994) Apoptosis in cancer therapy: crossing the threshold. *Cell* **78**: 539–542

Fisher TC, Milner AE, Gregory CD, Jackman AL, Aherne GW, Hartley JA, Dive C and Hickman JA (1993) bcl-2 modulation of apoptosis induced by anticancer drugs: resistance to thymidylate stress is independent of classical resistance pathways. *Cancer Res* **53**: 3321–3326

Fujii S, Ikenaka K, Fukushima M and Shirasaka T (1978) Effect of uracil and its derivatives on antitumour activity of 5-fluorouracil and 1-(2-tetrahydrofuryl)-5-fluorouracil. *Gann* **69**: 763–772

Fujii S, Kitano S, Ikenaka K and Shirasaka T (1979) Effect of coadministration of uracil or cytosine on the anti-tumour activity of clinical doses of 1-(2-tetrahydrofuryl)-5-fluorouracil and level of 5-fluorouracil in rodents. *Gann* **70**: 209–214

Gavrieli Y, Sherman Y and Ben-Sasson SA (1992) Identification of programmed cell death in situ via specific labeling of nuclear DNA fragmentation. *J Cell Biol* **119**: 493–501

Ikenaka T, Shirasaka T, Kitano S and Fujii S (1979) Effect of uracil on metabolism of 5-fluorouracil in vitro. *Jpn J Cancer Res (Gan)* **70**: 353–359

Kerr JF and Searle J (1972) A suggested explanation for the paradoxically slow growth rate of basal-cell carcinomas that contain numerous mitotic figures. *J Pathol* **107**: 41–44

Kerr JF, Wyllie AH and Currie AR (1972) Apoptosis: a basic biological phenomenon with wide-ranging implications in tissue kinetics. *Br J Cancer* **26**: 239–257

Kondo S, Barna BP, Kondo Y, Tanaka Y, Casey G, Liu J, Morimura T, Kaakaji R, Peterson JW, Werbel B and Barnett GH (1996) WAF1/CIP1 increases the susceptibility of p53 non-functional malignant glioma cells to cisplatin-induced apoptosis. *Oncogene* **13**: 1279–1285

Lichter AS and Lawrence TS (1995) Recent advances in radiation oncology. *New Engl J Med* **332**: 371–379

Lowe SW, Ruley HE, Jacks T and Housman DE (1993) p53-dependent apoptosis modulates the cytotoxicity of anticancer agents. *Cell* **74**: 957–967

Maehara Y, Kusumoto T, Kusumoto H, Anai H and Sugimachi K (1988) UFT is more antineoplastic against gastric carcinoma than 5-fluorouracil, 1-(2-tetrahydrofuryl)-5-fluorouracil and 1-hexylcarbamoyl-5-fluorouracil. *Chemotherapy* **34**: 484–489

Maehara Y, Sugimachi K, Akagi M, Kakegawa T, Shimazu H and Tomita M (1992) Early postoperative chemotherapy following noncurative resection for patients with advanced gastric cancer. *Br J Cancer* **65**: 413–416

Maehara Y, Takeuchi H, Oshiro T, Takahashi I, Inutsuka S, Baba H, Kohnoe S and Sugimachi K (1994) Effect of gastrectomy on the pharmacokinetics of tegafur, uracil, and 5-fluorouracil after oral administration of a 1:4 tegafur and uracil combination. *Cancer Chemother Pharm* **33**: 445–449

Meyn RE, Stephens LC, Hunter NR and Milas L (1994) Induction of apoptosis in murine tumours by cyclophosphamide. *Cancer Chemother Pharm* **33**: 410–414

Meyn RE, Stephens LC, Hunter NR and Milas L (1995*a*) Apoptosis in murine tumours treated with chemotherapy agents. *Anti-Cancer Drugs* **6**: 443–450

Meyn RE, Stephens LC, Hunter NR and Milas L (1995*b*) Kinetics of cisplatin-induced apoptosis in murine mammary and ovarian adenocarcinomas. *Int J Cancer* **60**: 725–729

Muggia FM, Wu X, Spicer D, Groshen S, Jeffers S, Leichman CG, Leichman L and Chan KK (1996) Phase I and pharmacokinetic study of oral UFT, a combinatin of the 5-fluorouracil prodrug tegafur and uracil. *Clin Cancer Res* **2**: 1461–1467

Ota K, Taguchi T and Kimura K (1988) Report on nationwide pooled data and cohort investigation in UFT phase II study. *Cancer Chemother Pharm* **22**: 333–338

Pazdur R, Lassere Y, Rhodes V, Ajani JA, Sugarman SM, Patt YZ, Jones Jr. DV, Markowitz AB, Abbruzzese JL, Bready B and Levin B (1994) Phase II trial of uracil and tegafur plus oral leucovorin: an effective oral regimen in the treatment of metastatic colorectal carcinoma. *J Clin Oncol* **12**: 2296–2300

Sakaguchi Y, Stephens LC, Makino M, Kaneko T, Strebel FR, Danhauser LL, Jenkins GN and Bull JM (1994) Apoptosis in normal tissues induced by 5-fluorouracil: comparison between bolus injection and prolonged infusion. *Anticancer Res* **14**: 1489–1492

Schumacher HJ, Wilson JG and Jordan RL (1969) Potentiation of the teratogenic effects of 5-fluorouracil by natural pyrimidines. II. Biochemical aspects. *Teratology* **2**: 99–105

Strobel T, Swanson L, Korsmeyer S and Cannistra SA (1996) BAX enhances paclitaxel-induced apoptosis through a p53-independent pathway. *Proc Natl Acad Sci USA* **93**: 14094–14099

Toide H, Akiyoshi H, Minato Y, Okuda H and Fujii S (1977) Comparative studies on the metabolism of 2-(tetrahydrofuryl)-5-fluorouracil and 5-fluorouracil. *Gann* **68**: 553–560

Vasey PA, Jones NA, Jenkins S, Dive C and Brown R (1996) Cisplatin, camptothecin, and taxol sensitivities of cells with p53-associated multidrug resistance. *Mol Pharmacol* **50**: 1536–1540

Wyllie AH (1992) Apoptosis and the regulation of cell numbers in normal and neoplastic tissues: an overview. *Cancer Metastasis Rev* **11**: 95–103

© Cancer Research Campaign 1998